# Vaginal bleeding as a sign of Crimean–Congo hemorrhagic fever infection: a case report

**DOI:** 10.1186/s13256-022-03303-z

**Published:** 2022-02-22

**Authors:** Shohra Qaderi, Hossein Hatami, Ahmad Murad Omid, Jalal Sayad

**Affiliations:** 1 Student Research Committee, Shahid Beheshti University of Medical Sciences, Tehran, Iran; 2 Center of Public Health, Environmental and Occupational Hazards Control, Shahid Beheshti University of Medical Science, Tehran, Iran; 3 Crimean-Congo Hemorrhagic Ward, Department of Communicable Disease, Kabul Antani Hospital, Ministry of Public Health, Kabul, Afghanistan

**Keywords:** Uterine bleeding, Afghanistan, Crimean–Congo hemorrhagic fever (CCHF)

## Abstract

**Background:**

Crimean–Congo hemorrhagic fever is a severe vector-borne viral hemorrhagic fever with considerable mortality in humans. This disease is endemic in Afghanistan, and its incidence rate has rapidly increased in recent years. This infection can cause a broad range of hemorrhage manifestations including epistaxis, petechial or purpuric rashes, hematemesis, and melena; however, vaginal bleeding is also reported as a rare manifestation.

**Case presentation:**

We report the case of a previously healthy 30-year-old Afghan female of shepherding occupation, with a sudden onset of fever, generalized body pain, epistaxis, and vaginal bleeding. She was admitted to the hospital after 7 days of symptom manifestation, with predominant signs being high fever, vaginal bleeding, and elevated liver enzymes. The serological test result for Crimean–Congo hemorrhagic fever was positive. She was treated with oral ribavirin and discharged with normal parameters.

**Conclusions:**

People in high-risk professions in endemic areas should be informed that vaginal bleeding is a serious symptom and requires immediate action and, therefore, might be attributed to nongynecologic disorders.

## Introduction

Crimean–Congo hemorrhagic fever (CCHF), is an acute, severe vector-borne viral hemorrhagic fever with considerable mortality in humans. The causative agent is the genus *Nairovirus* within the family of Bunyaviridae [1, 2]. Human CCHF is characterized by a sudden onset of fever, headache, dizziness, nausea, weakness, irritability, and severe limb and loin pain, which has shown a wide range of severity, from mild, nonspecific febrile illness to shock and death. The hemorrhagic symptoms include petechiae and/or ecchymosis spreading over the chest, abdomen, and rest of the body; sometimes large purpuric areas are reported. In severe cases, bleeding from the gums, nose, lung, uterus, and intestine are reported and can progress to multiorgan failure [3, 4]. The case fatality rate is about 10–40% [5]. The disease is endemic in more than 30 countries worldwide. In recent years, the incidence of CCHF has increased rapidly in the countries of World Health Organization Eastern Mediterranean Region (WHO EMR), with sporadic human cases and outbreak of CCHF being reported for a number of countries in the region [6, 7]. Afghanistan is located in the region in which *Hyalomma* ticks are quite common, and experiences the disease regularly; an average of 5–50 human cases are reported every year in the country. The highest numbers of cases were reported in Kabul and Herat provinces, which share borders with Pakistan and Iran, where livestock movement across borders is not controlled. According to the latest related report in 2017, the total number of cases reported in Afghanistan was 245 [48% case fatality rate (CFR)], with one case of vaginal bleeding due to CCHF [8, 9]. Thus, in this case report, we present the epidemiological, clinical, and paraclinical features of Crimean–Congo hemorrhagic fever in patients with vaginal bleeding as a rare and nongynecological symptom who were admitted to Antani Hospital in Kabul, Afghanistan in 2018.

## Case presentation

On the evening of 5 May 2018 (*T* = 0), a non-Kabul resident, 30-year-old Afghan single female was admitted to Kabul Antani Hospital because of fever, pain, hemorrhage (petechia, epistaxis, and uterine bleeding), and vomiting. The patient reported sudden-onset fever and generalized body ache 1 week prior to hospital presentation (*T* = 7). Two days later, the patient had nausea and vomiting regardless of having meals; however, she had not been brought to any health centers. One day later (*T* = 3), she presented epistaxis and vaginal bleeding [last menstrual period (LMP) was on 20 April]. Then, she was brought to the hospital and admitted to the CCHF ward of Kabul Antani Hospital. She denied history of travel, tick bite, and sick contacts; she had no prior medical conditions or medication use. She did not consume alcohol or smoke. She lived in a rural area, and all her family members worked as shepherds. At the time of admission, pulse rate was 92 beats per minute, blood pressure 70/50 mmHg, and respiratory rate 20 breaths per minute, and she had a temperature of 38 °C. Physical examination findings included pallor, with petechiae and uterine bleeding (without gynecological history). The patient appeared confused on examination. The patient was anemic with a remarkably low platelet count 10 × 10^3^/μL. The levels of aspartate transaminase (AST) and alanine aminotransferase (ALT) were 1921 U/L and 1318 U/L, respectively, alkaline phosphatase (ALP) was 796 U/L, and other laboratory test results are presented in Table 1. CCHF virus was detected from a blood sample drawn on the fourth day with a positive enzyme-linked immunosorbent assay (ELISA) test for specific CCHF immunoglobulin M (IgM) antibodies by the National Laboratory of Public Health Ministry. Results of hepatitis B testing (hepatitis B surface antigen, hepatitis B core IgG antibody) and hepatitis C testing (the HCV antibody test) were negative. Owing to financial issues, some serological tests such as *Leptospira*, *Salmonella*, *Toxoplasma* species, and Lyme disease were not performed. In the CCHF department of the hospital, the patient had been prescribed oral ribavirin as recommended by the World Health Organization (WHO), and supportive therapy was given as presented in Table 2. The patient was hospitalized for a total of 8 days. Details of the development of vital signs over the course of receiving ribavirin during her hospitalization are presented in Table 3. As patients are not normally followed up in governmental hospitals of Afghanistan, information about her condition after discharge was not available.

**Table 1:** Laboratory data*

Variables	Reference range	Hospital range
Blood		
Hemoglobin (g/dl)	12–15	10
Hematocrit (%)	36–45	38.4
White-cell count (per mm^3^)	4–11	2300
Platelet count (per mm^3^)	150–400	10,000
Alanine aminotransferase (U/L)	0–40	1318
Aspartate aminotransferase (U/L)	0–40	1921
Total bilirubin (mg/dl)	< 1.2	2.2
Direct bilirubin (mg/dl)		1
Indirect bilirubin (mg/dl)^β^		1.2
Prothrombin-time international normalized ratio	0.9–1.1	3
Urine		
Color	Yellow	Dark
Clarity	Clear	
Specific gravity	1.033	1.000
pH	7	7.2
Protein		2+
White cells per high-power field		_2+_
Red cells per high-power field		_3+_

*All these tests were carried out in Antani’s hospital laboratory.

mg/dl: Milligram Per Decilitre

^β^We obtained indirect bilirubin via total bilirubin − direct bilirubin

**Table 2:** Treatment*

Intervention	Dose/frequency	First day	Second day	Third day	Fourth day	Fifth day	Sixth day	Seventh day	Eighth day
Pantoprazole	40 mg once	Yes	Yes	Yes	Yes	Yes	No	No	No
Ringer lactate	1000 cc once	Yes	Yes	Yes	Yes	Yes	Yes	Yes	Yes
Cefotaxime	2 g three times daily	Yes	Yes	Yes	Yes	Yes	Yes	Yes	Yes
Ribavirin	2000 mg/once	Yes	Yes	Yes	Yes	Yes	Yes	Yes	Yes
Platelet mass	400 cc once	Yes	Yes	Yes	Yes	Yes	Yes	Yes	Yes

*Treatment received by patient over 8 days of hospitalization. Ribavirin 400 mg daily for two more 2 days and cefotaxime 1 g daily were prescribed as home treatment

**Table 3:** Daily vital signs

Days of admission	First	Second	Third	Fourth	Fifth	Sixth	Seventh	Eighth
Blood pressure (systole/diastole, mmHg)	70/50	87/50	100/60	110/70	100/60	100/70	110/60	110/70
RR (per minute)	20	20	20	20	22	20	22	20
PR (per minute)	78	92	88	82	80	84	74	74
T (°C)	38	37.6	37.4	37.2	37.3	37	37	37

*RR* respiratory rate, *PR* pulse rate. *T* temperature

## Discussion

Here, we presented the case of a woman working as a shepherd who had uterine bleeding as a rare and nongynecological sign of CCHF that led to 8 days of hospitalization. Crimean–Congo hemorrhagic fever is a severe tick-borne disease with a wide range of geographical distribution. The disease is endemic over a wide geographic area, mainly in Southern Europe, Africa, the Middle East, and Asia [3]. Afghanistan is located in the endemic region of this disease and experiences the disease regularly, about 5–50 cases every year, but the majority of cases occur around Eid-Adha (sacrifice feast in Islam) in which there is increased human contact with livestock due to sacrificial rituals/norms. Residence in rural area and close contact with livestock are shown to be more common in CCHF cases, both of which were the case in our patient. These groups should be considered as susceptible/vulnerable to CCHF infection. There is little information about the pathogenesis of the infection, but the interaction between the virus and host cells (mainly endothelial cells and immune cells) is most likely responsible for the pathogenesis. There are two theories about CCHF pathogenesis: the first is that the virus interacts with endothelial cells, and the latter is that the virus interacts indirectly with immune cells, which results in the release of mediators [10]. Hemorrhage as a main sign of CCHF has two mechanisms: the first is through a direct effect on hemostasis cells such as thrombocytes and endothelial cells, and the second is through immune and inflammatory pathways that lead to cellular damage. The liver is the main site of CCHF virus replication. During replication, the virus is able to induce cytopathic effects, including hepatocyte necrosis and apoptosis, Kupffer cell hyperplasia, and fatty changes [6–10]. The majority of Afghanistan CCHF cases visited hospitals at the hemorrhagic phase of the infection, and this delay reduces the efficacy of ribavirin and leads to more serious health complications [8, 11]. This febrile, hemorrhage disease can cause a broad range of hemorrhagic manifestations including petechiae and/or ecchymosis spreading over the chest, abdomen, and rest of the body; sometimes large purpuric areas are reported. In severe cases, bleeding from the gums, nose, lung, uterus, and intestine are reported and can progress to multiorgan failure; however, vaginal bleeding is a rare manifestation of CCHF infection. According to previous studies, the hemorrhagic phase of the infection starts between the third and sixth day of symptom onset; in our patient, bleeding started on day 4 [6]. Statistical significance has not been reported for age, gender, and tick contact, regarding fatality to the infection [14]. Hemorrhage has been reported in all fatal cases, and the presence of bleeding is the most important predictive factor of fatality. Epistasis is the most common type of bleeding, and bleeding in more than one system has been seen in patients who died, but it is difficult to determine which types of hemorrhages are associated with fatality of this infection [12, 14]. Previous studies investigated vaginal bleeding as a sign of CCHF infection [9–14]. Thrombocytopenia, leukopenia, and elevation of AST, ALT, and international normalized ratio (INR) are some typical hematological changes in a patient with CCHF infection, which were seen in our case. The elevation of AST and INR and thrombocytopenia were found to be predictors of fatality/mortality [14]. This case report, however, puts an emphasis on the importance of vaginal bleeding being considered a matter of urgency that requires immediate action and, therefore, might be attributed to nongynecologic disorders.

## Conclusion

CCHF is a serious public health problem/threat that is endemic in Afghanistan. This infection can cause a wide range of hemorrhage manifestations of which vaginal bleeding is a serious symptom that requires immediate action. Vaginal bleeding is an uncommon symptom of this disease and, therefore, might be attributed to nongynecologic disorders. Low platelet count, low level of white blood cells, and vaginal bleeding, especially in endemic areas, could suggest CCHF infection.

## Data Availability

Not applicable. No new data generated.

## Abbreviation

CCHF: Crimean–Congo hemorrhagic fever
